# Combined intramuscular and intraspinal transplant of bone marrow cells improves neuromuscular function in the SOD1^G93A^ mice

**DOI:** 10.1186/s13287-020-1573-6

**Published:** 2020-02-07

**Authors:** Anna Martínez-Muriana, Diego Pastor, Renzo Mancuso, Amaya Rando, Rosario Osta, Salvador Martínez, Rubèn López-Vales, Xavier Navarro

**Affiliations:** 1 Institute of Neurosciences and Department of Cell Biology, Physiology and Immunology, Universitat Autònoma de Barcelona, Barcelona, Spain; 2 Centro de Investigación Biomédica en Red sobre Enfermedades Neurodegenerativas (CIBERNED), Bellaterra, Spain; 3 Centro de Investigación Deportiva, Universidad Miguel Hernández, Elche, Spain; 4 Instituto de Neurociencias, UMH-CSIC, San Juan de Alicante, Spain; 5 VIB Center for Brain and Disease Research, KU Leuven, Leuven, Belgium; 6 Laboratory of Genetic Biochemistry (LAGENBIO), Health Research Institute of Aragón, Universidad de Zaragoza, Zaragoza, Spain; 7 Faculty of Medicine, Universitat Autònoma de Barcelona, 08193 Bellaterra, Spain

**Keywords:** ALS, SOD1, Bone marrow, Stem cells, Motoneuron disease

## Abstract

**Background:**

The simultaneous contribution of several etiopathogenic disturbances makes amyotrophic lateral sclerosis (ALS) a fatal and challenging disease. Here, we studied two different cell therapy protocols to protect both central and peripheral nervous system in a murine model of ALS.

**Methods:**

Since ALS begins with a distal axonopathy, in a first assay, we performed injection of bone marrow cells into two hindlimb muscles of transgenic SOD1^G93A^ mice. In a second study, we combined intramuscular and intraspinal injection of bone marrow cells. Fluorescence-activated cell sorting was used to assess the survival of the transplanted cells into the injected tissues. The mice were assessed from 8 to 16 weeks of age by means of locomotion and electrophysiological tests. After follow-up, the spinal cord was processed for analysis of motoneuron survival and glial cell reactivity.

**Results:**

We found that, after intramuscular injection, bone marrow cells were able to engraft within the muscle. However, bone marrow cell intramuscular injection failed to promote a general therapeutic effect. In the second approach, we found that bone marrow cells had limited survival in the spinal cord, but this strategy significantly improved motor outcomes. Moreover, we also found that the dual cell therapy tended to preserve spinal motoneurons at late stages of the disease and to reduce microgliosis, although this did not prolong mice survival.

**Conclusion:**

Overall, our findings suggest that targeting more than one affected area of the motor system at once with bone marrow cell therapy results in a valuable therapeutic intervention for ALS.

## Introduction

Amyotrophic lateral sclerosis (ALS) is an adult-onset neurodegenerative disease characterized by a progressive and selective death of upper and lower motoneurons (MNs) [1]. Resulting from neuronal loss, patients suffer progressive atrophy and eventual muscle paralysis that leads to their death a few years after the onset [2]. As in other neurodegenerative disorders, most ALS cases are sporadic; however, several mutations have been identified in familial cases of ALS (fALS). The most prevalent forms include mutations encoded in the TARDBP (*TDP43*) gene, the hexanucleotide repeat expansions in chromosome-9 open reading frame 72 (*C9ORF72*) or in the superoxide dismutase gene (*SOD1*) [3]. Indeed, the most used experimental model of ALS is a transgenic mouse that overexpresses a high-copy number of the mutated form of the human *SOD1* gene [4]. This transgenic ALS model mimics the human pathology, being the most used preclinical tool to study the disorder and test novel therapies [5]. The pathogenic mechanisms underlying MN death involve alterations of multiple molecular mechanisms, including glutamate-mediated excitotoxicity, defective axonal transport, or activation of neighboring glial cells [6–8]. The simultaneous disruption of several molecular mechanisms and cell types raises the difficulty to find an effective treatment. In fact, novel therapies that only target a single factor have largely failed when translated into human clinical trials [9]. These failures suggest that more effective therapeutic approaches should act simultaneously on several targets in order to mediate global neuroprotection. In this way, cell therapy has emerged as a promising way to target several cells and mechanisms involved in ALS.

Bone marrow cells (BMCs) are easy and fast to isolate and do not need further manipulation before their transplant, thus being a very convenient source for cell therapy applications. BMCs are composed by several cell subsets that include hematopoietic progenitors and a heterogeneous population of mesenchymal stromal cells (MSCs) [10]. Several studies demonstrate that BMC transplantation ameliorates the progression of diverse neurodegenerative diseases, including ALS [11–13]. Similarly, previous reports from our group have revealed that intramuscular injections of BMCs in mdf/ocd and SOD1^G93A^ mice induce protection of spinal MNs and improve motor function [14, 15]. The exact molecular mechanisms underlying the beneficial effects of BMC transplantation are still under debate; however, they likely involve the immunomodulatory actions of BMCs and the release of neurotrophic factors [13]. Studies using BMCs as factor carriers have shown neuroprotection when transplanted into the spinal cord of SOD1^G93A^ mice and human patients [12, 16–19]. However, none of these treatments preserved neuromuscular function and stability of neuromuscular junctions (NMJs) at suitable levels [20]. Previous studies have attempted to protect both NMJs [15] and MNs in SOD1^G93A^ mice [12, 21, 22] separately, showing transient mild effects on disease outcomes. Since ALS begins with a distal axonopathy that precedes MN soma degeneration [23], in this work, we have attempted to protect both MN soma, as well as, their terminal axons in skeletal muscle. Thus, we investigated the effects of the combined injection of BMCs into hindlimb muscles and in the spinal cord to protect NMJs and spinal cord MNs, respectively, in SOD1^G93A^ mice.

## Materials and methods

### Animals

The animals used in this work were transgenic mice carrying the G93A human SOD1 mutation (B6SJL-Tg [SOD1-G93A]1Gur) obtained from the Jackson Laboratory (Bar Harbor, ME, USA) and provided from the colony maintained at the University of Zaragoza. For phenotyping, DNA was extracted from tail samples and further analyzed by PCR to identify hemizygous transgenic mice and wildtype littermates. Mice were maintained with food and water ad libitum at room temperature of 22 ± 2 °C under a 12:12-h light–dark cycle. Endpoint criterium was considered when animals lost the righting reflex for longer than 30 s. At 8 weeks of age (prior to BMC injection), animals were electrophysiologically tested to obtain baseline levels. SOD1^G93A^ females and males were then distributed among the different experimental groups according to their progenitors, weight, and electrophysiology baseline values in balanced groups, receiving either BMCs or vehicle.

### Bone marrow cells

BMCs were collected from female C57Bl6/J mice or GFP (green fluorescent protein) mutant mice (C57Bl/6-Tg [ACTB-EGFP]1Osb/J) [24]. Using the same procedures already described by our group [14, 15], femurs of these mice were harvested and the attached muscles and connective tissues were removed. The epiphyseal ends of the bones were clipped and the marrow was extracted using intraosseous perfusion with 2 ml of Dulbecco’s modified Eagle medium (DMEM, Gibco). Immediately, BMCs were centrifuged at 1000 rpm at 4 °C for 5 min and concentrated to 35,000 cells/μl. For the identification of the cells after injection, we used GFP-labeled BMCs for long-term studies or labeled with PKH67 (Sigma), a cell membrane labeling dye, for the short-term survival experiments.

For transplantation, 8-week-old, prior to MN death and when axonal degeneration starts [25], SOD1^G93A^ mice were anesthetized with ketamine (90 mg/kg, i.p.) and xylazine (10 mg/kg, i.p.). As described previously by our group [14, 15], for intramuscular injections, a total volume of 14 μl green-labeled BMC suspension (0.5 × 10^6^ BMCs) was injected into quadriceps femoris (QF) and 7 μl (0.25 × 10^6^ BMC) into tibialis anterior (TA) muscles bilaterally using a 27-G needle attached to a Hamilton syringe. For intraspinal injections, L4-L5 spinal cord segments were exposed by means of a small laminectomy and a volume of 5 μl was injected using a glass needle (30 μm i.d.) coupled to a 10-μl Hamilton syringe. Injections were made at a perfusion speed of 2 μl/min controlled by an automatic injector (KDS 310 Plus; KD Scientific), and the tip of the needle was maintained inside the tissue 3 min after each injection to avoid liquid reflux. Sham-treated SOD1^G93A^ mice received the same volume injections of vehicle (DMEM).

### Functional evaluation

Locomotion, coordination, and muscle strength was evaluated by rotarod test. Prior to cell injections, all animals were trained twice per week on the rotating rod at a constant speed of 14 rpm. All animals used in this study reached baseline levels (an arbitrary time of 180 s). After injections, BMC- or sham-treated SOD1^G93A^ mice were evaluated in the rotarod once per week until 16 weeks of age. The time each animal was able to remain on the rotarod turning at a constant speed of 14 rpm was measured.

Locomotion was further analyzed using a speed-controlled treadmill (Digigait, Mouse Specifics). To evaluate the maximum speed that SOD1^G93A^ mice were able to run at the end-stage of the disease, animals were placed on the treadmill belt and their capacity to run with increasing velocities (5, 10, 15, 20, 25, and 30 cm/s) was recorded [26].

Electrophysiological tests were used to evaluate motor function every 2 weeks from 8 to 16 weeks of age. Nerve conduction test was performed by stimulating the sciatic nerve (square pulses of 20-μs duration, Grass S88) through a pair of needles located percutaneously in the sciatic notch. Compound muscle action potentials (CMAPs) were recorded in sciatic nerve muscle targets: TA and gastrocnemius medialis muscle (GM) with microneedle electrodes placed on the muscle [25]. All recordings were amplified and visualized in a digital oscilloscope (Tektronix 450S). Measurements of the CMAP amplitude were obtained from the baseline to the maximal negative peak and the latency from stimulus to the onset of the first negative deflection. The recording needles were placed under a microscope, guided by anatomical landmarks, to ensure reproducibility of needle location on all animals. During the tests, the mouse body temperature was maintained using a controlled heating pad.

### Histology

At 16 weeks of age, BMC or SHAM SOD1^G93A^ and wildtype littermate mice were euthanized with an overdose of sodium pentobarbital. Immediately, animals were transcardially perfused with a solution of 4% paraformaldehyde (PFA) in 0.1 M phosphate buffer (PB) and tissue samples were harvested. Treated and control muscles were cryopreserved and maintained in a solution of 30% sucrose with azide in 0.1 M PB at 4 °C. Spinal cord lumbar segments were harvested and post-fixed in 4% PFA for 2 h, and then cryopreserved and maintained at 4 °C with 30% sucrose and azide in 0.1 M PB.

Lumbar spinal cords were serially cut in transverse sections of 40-μm thickness using a cryostat (Leica). Free-floating series of 10 sections were sequentially collected and kept in Olmos solution at − 20 °C. To assess MN survival, one series was rehydrated and stained for 3 h with a 3.1 mM Cresyl violet solution. Slices were then washed, dehydrated, and mounted with DPX (Fluka). L4-L5 MNs were localized by morphology and size. Only MNs that were polygonal-shaped, larger than 20 μm in diameter and with well-stained nucleoli were counted. MNs present in the lateral side of both ventral horns were quantified in four serial sections of the L4-L5 segment [25].

For immunostainings, slides were placed over a hotplate at 37 °C for 30 min. Then, samples were rehydrated in PBS for 5 min and further blocked with a blocking solution of 5% NDS in PBS-T 0.1% at room temperature. Once blocked, sections were incubated overnight at 4 °C with anti-Iba1 (Wako, 1:1000) and anti-GFAP (Dako, 1:1000). Sections were then washed in PBS-T and further incubated with a specific secondary antibody bound to an Alexa-594 or -488 fluorocrom (1:500, Invitrogen) for 1 h.

### Flow cytometry

Fluorescence-activated cell sorting (FACS) was used to characterize BMCs and to assess the survival of the transplanted cells into the target tissues. For the immunophenotyping, BMCs were obtained from femurs of C57Bl6 mice as indicated above. Then, red blood cells (RBC) were lysed using RBC lysis Buffer (10x) following the manufacturer’s instructions (Biolegend, 420301). Cell suspension was further washed with FACS buffer (DMEM + 10% FBS) and centrifuged twice at 300×*g* for 10 min at 4 °C. Samples were further split in several tubes and immunostained. Primary antibody labeling was performed for 1 h at 4 °C in FACS buffer with the following conjugated antibodies: mouse lineage antibody cocktail (Lin)-PerCP-Cy5.5 (1:200, 561317, BD Biosciences), Sca-1-PE-Cy7 (1:200, 561021, BD Biosciences), c-Kit-APC (1:200, 561074, BD Biosciences), and CD34 (1:200, 560238, BD Biosciences). For the gating strategy, we set up the cut off based on the corresponding isotypes control expression (Additional file 1: Figure S1A). For the analysis, cells were first gated based on size (FSC) and complexity (SSC) to remove all the debris. Cells were further gated based on Lin expression to discriminate between progenitors (Lin^−^) and lineage mature cells (Lin^+^). The percentage of mesenchymal stromal cells (MSC) was calculated by counting all the Lin^−^, Sca-1^+^, c-Kit^−^ and CD34^−^ cells [27].

For the in vivo evaluation of BMC engraftment, animals were sacrificed and transcardially perfused with 60 ml of 0.9% sterile saline solution, and spinal cords and hindlimb muscles were rapidly collected and kept in DMEM. Samples were further mechanically broken up using a syringe and passed through a 40-μm cell strainer (BD2 Falcon) with DMEM to disaggregate the cells. To eliminate debris, the obtained cell suspension was centrifuged twice at 300×*g* for 10 min at 4 °C. Centrifuged cells were further washed, and the obtained pellet was suspended and fixed in a 1% PFA solution and immediately analyzed in a FACSCanto flow cytometer (BD Biosciences). Grafted cells were distinguished from tissue resident cells using the FITC channel where both GFP and PKH67 dyes are detected.

### Statistics

Data are represented as mean ± SEM and analyzed using the GraphPad Prism 6 software package (GraphPad Software). Locomotion and electrophysiological results were analyzed using two-way repeated measurements ANOVA with Tukey post-hoc test for multiple comparisons. For histological data, two-tailed *t*-Student test was used. Survival and forced locomotion results were analyzed using the Mantel–Cox test. Differences were considered statistically significant at *p* < 0.05.

## Results

### Bone marrow cells immunophenotypic characterization

**Fig. 1:**
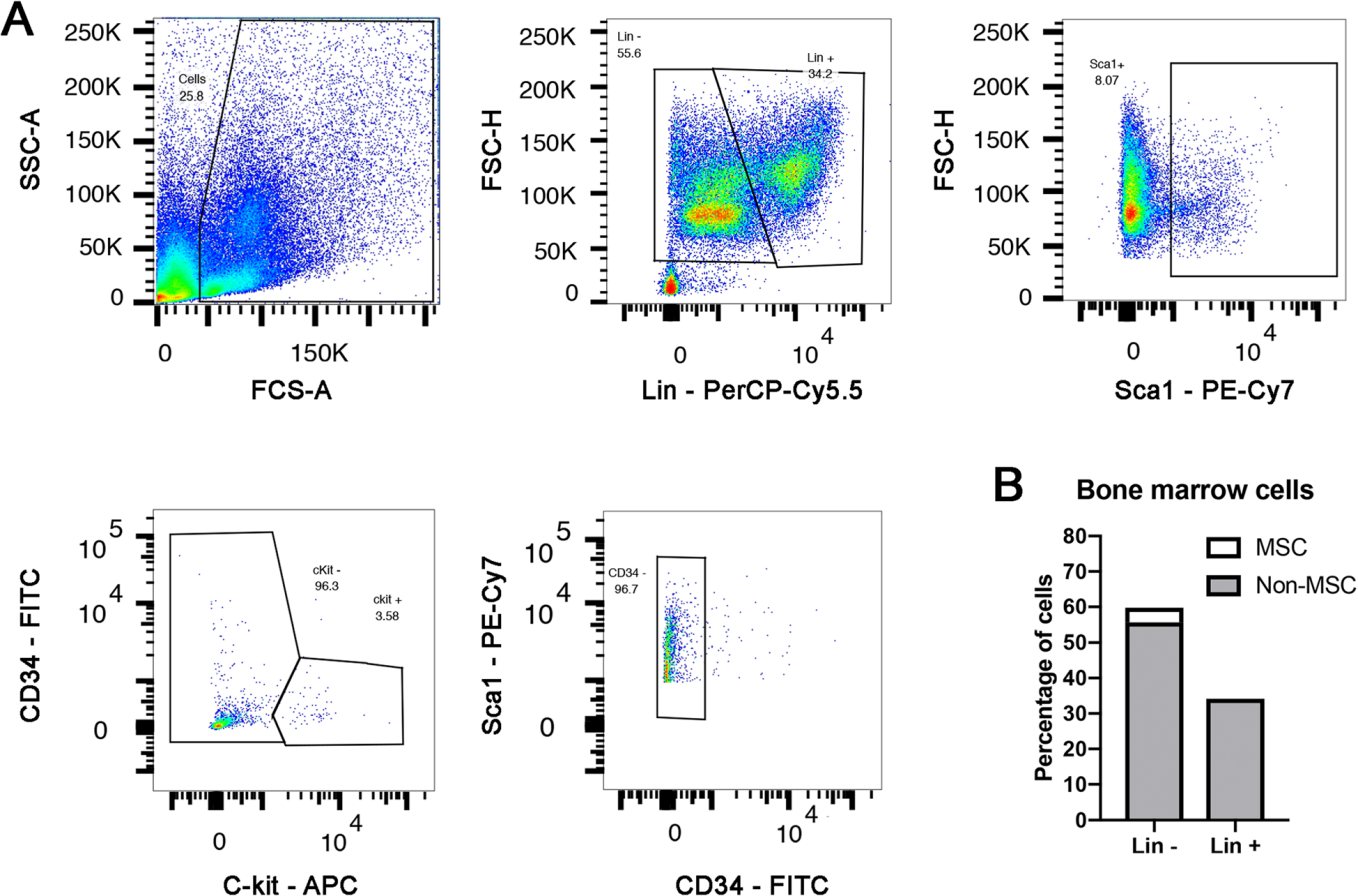
Bone marrow cells FACS characterization. **a** Representative FACS plots showing the gating strategy to quantify the amount of hematopoietic lineage cells (Lin^+^), the precursor cells (Lin^−^) and the MSCs (Lin^−^, Sca-1^+^, c-Kit^−^, CD34^−^). **b** Bar plot showing the percentage of each population within the mouse bone marrow

Adult mouse bone marrow is composed by two main stem cell lineage populations: hematopoietic stem cells (HSCs) and mesenchymal stromal cells (MSCs). Previous reports have demonstrated MSCs therapeutic properties in neurodegenerative diseases, including ALS [13]. However, the MSC population in the mouse bone marrow is quite limited. Here, using FACS immunophenotyping, we found that mouse bone marrow is mainly composed by mature lineage hematopoietic cells (~ 34%, Lin^+^) and progenitor cells (~ 55%, Lin^−^) (Fig. 1a, b). MSCs (Lin^−^, Sca-1^+^, c-Kit^−^ and CD34^−^), which contain an heterogenous population of progenitors and stem cells [10], only represented ~ 4% of the whole bone marrow content (Fig. 1a, b).

### BMCs do not improve neuromuscular function in grafted muscle of SOD1^G93A^ mice

**Fig. 2:**
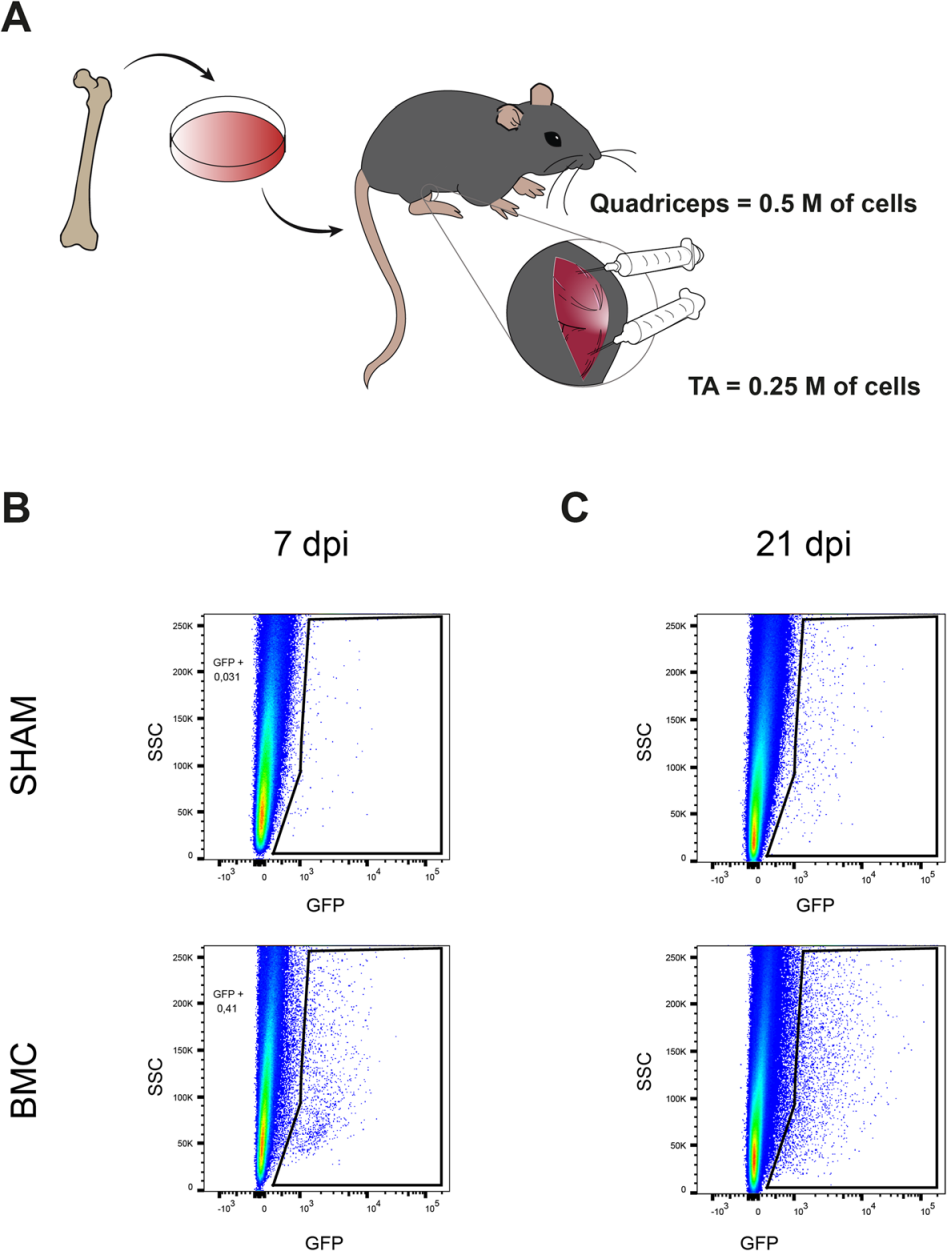
Bone marrow cells survival after transplantation in hindlimb muscles of SOD1^G93A^ mice. **a** Representative drawing showing the first study design. **b**, **c** Representative FACS plots showing green-labeled cells in muscle at 7 days (**b**) and 21 days (**c**) after BMC injection in the tibialis anterior (TA) muscle

Prior to assess the therapeutic effect of bone marrow cell injection, we evaluated the survival of the cell grafting within the muscle (Fig. 2a). We injected bone marrow green-labeled cells in hindlimbs muscles of SOD1^G93A^ mice and performed flow cytometry at 7 and 21 days post-injection (dpi). FACS analysis revealed that green-labeled BMCs were engrafted within the TA muscle for at least 3 weeks post-injection (Fig. 2b, c).

**Fig. 3:**
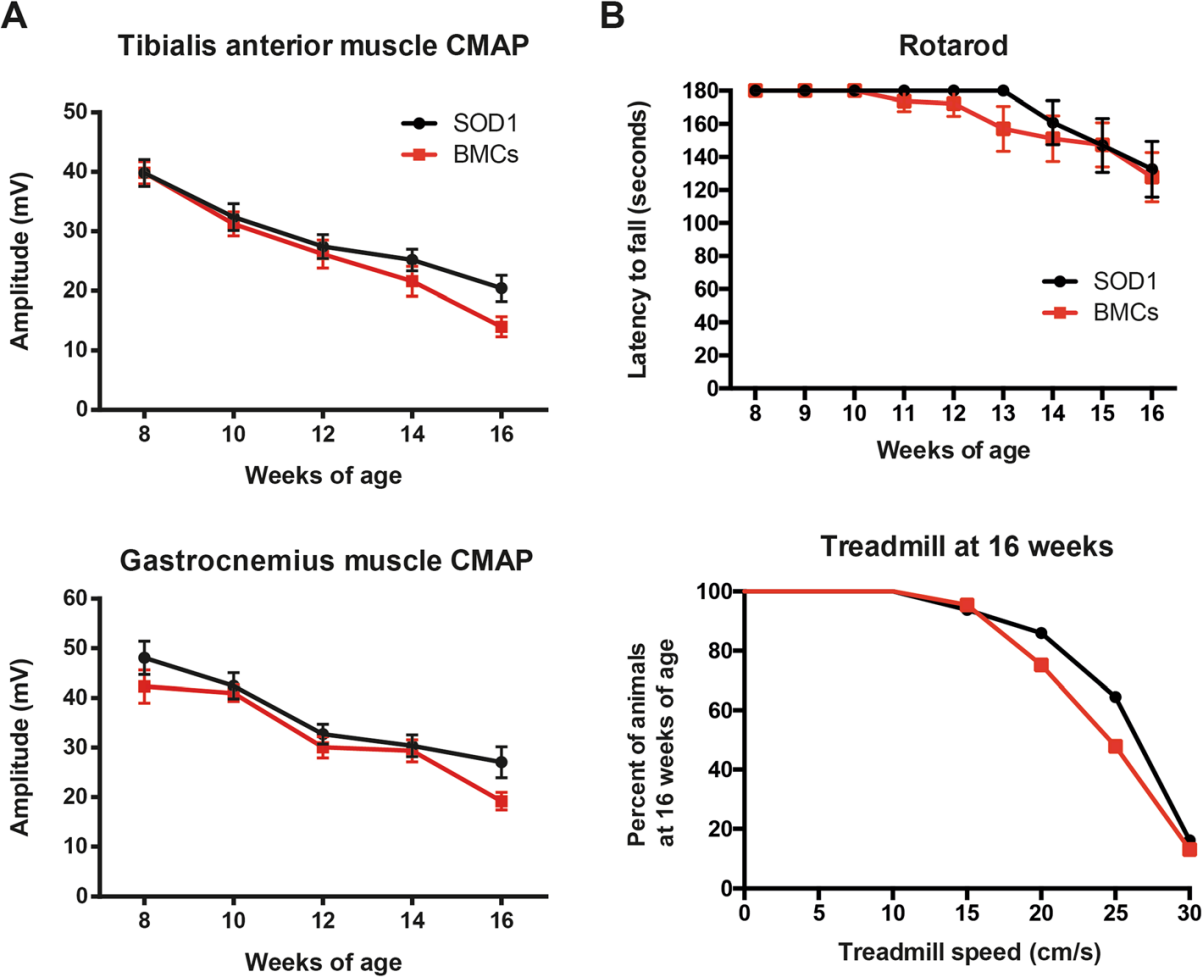
Effects of bone marrow cells injection in hindlimb muscles of SOD1^G93A^ mice. **a** Electrophysiological analysis of BMC injection effect in treated muscles (tibialis anterior) and non-treated muscles (Gastrocnemius medialis) (*n* = 16 SHAM SOD1^G93A^ vs. 17 BMC-treated SOD1^G93A^). **b** Gait and locomotion analysis by rotarod (*n* = 15 SHAM SOD1^G93A^ vs. 16 BMCs-treated SOD1^G93A^) and forced locomotion test (*n* = 8 SHAM SOD1^G93A^ vs. 11 BMCs-treated SOD1^G93A^) after injection of BMCs in hindlimb muscles. Error bars indicate SEM

**Fig. 4:**
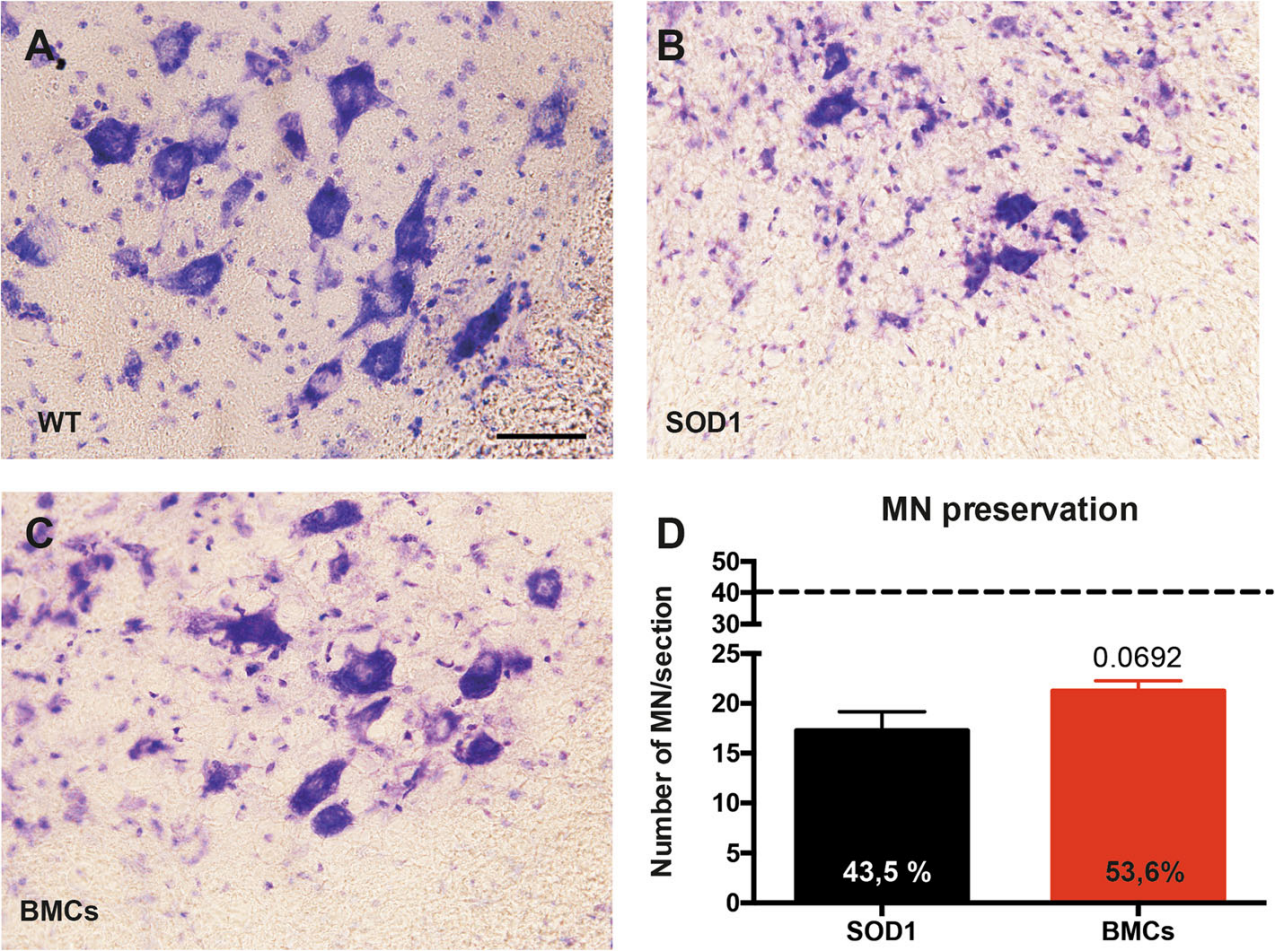
Analysis of MN survival after injection of BMCs in hindlimb muscles of SOD1^G93A^ mice. **a**–**c** Representative micrographs showing lumbar spinal MNs in **a** WT, **b** SHAM SOD1, or **c** BMC-treated SOD1 mice. Scale bar = 50 μm. **d** Quantification of MN sparing after treatment with BMCs (*n* = 7 SHAM SOD1^G93A^ vs. 9 BMCs-treated SOD1^G93A^). Dotted line indicates the average number of MNs in WT mice. Error bars indicate SEM

Once determined that BMCs were able to survive in the muscle, we tested their therapeutic effects in the SOD1^G93A^ mouse model of ALS. Bilateral injections of BMCs or vehicle were performed in the TA and QF muscles of SOD1^G93A^ mice at 8 weeks of age (Fig. 3a). Electrophysiological tests showed that BMC injection did not improve the CMAP amplitude compared to the DMEM-injected animals, neither in grafted muscles (TA) nor in non-injected gastrocnemius medialis (GM) muscles, used as internal control. Besides, bilateral BMC injections were not enough to improve the overall motor outcome, neither in the rotarod nor in the treadmill tests (Fig. 3b). Then, we aimed to know whether BMC intramuscular injections were able to protect spinal MNs, as reported in a previous study [14]. We found that BMC-treated animals had 10% higher number of surviving MNs compared to SHAM mice, although this difference did not reach statistical significance (*p* = 0.0692) (Fig. 4a–d).

### Intraspinal and intramuscular BMC injections preserve lower motoneuron function in SOD1^G93A^ mice

**Fig. 5:**
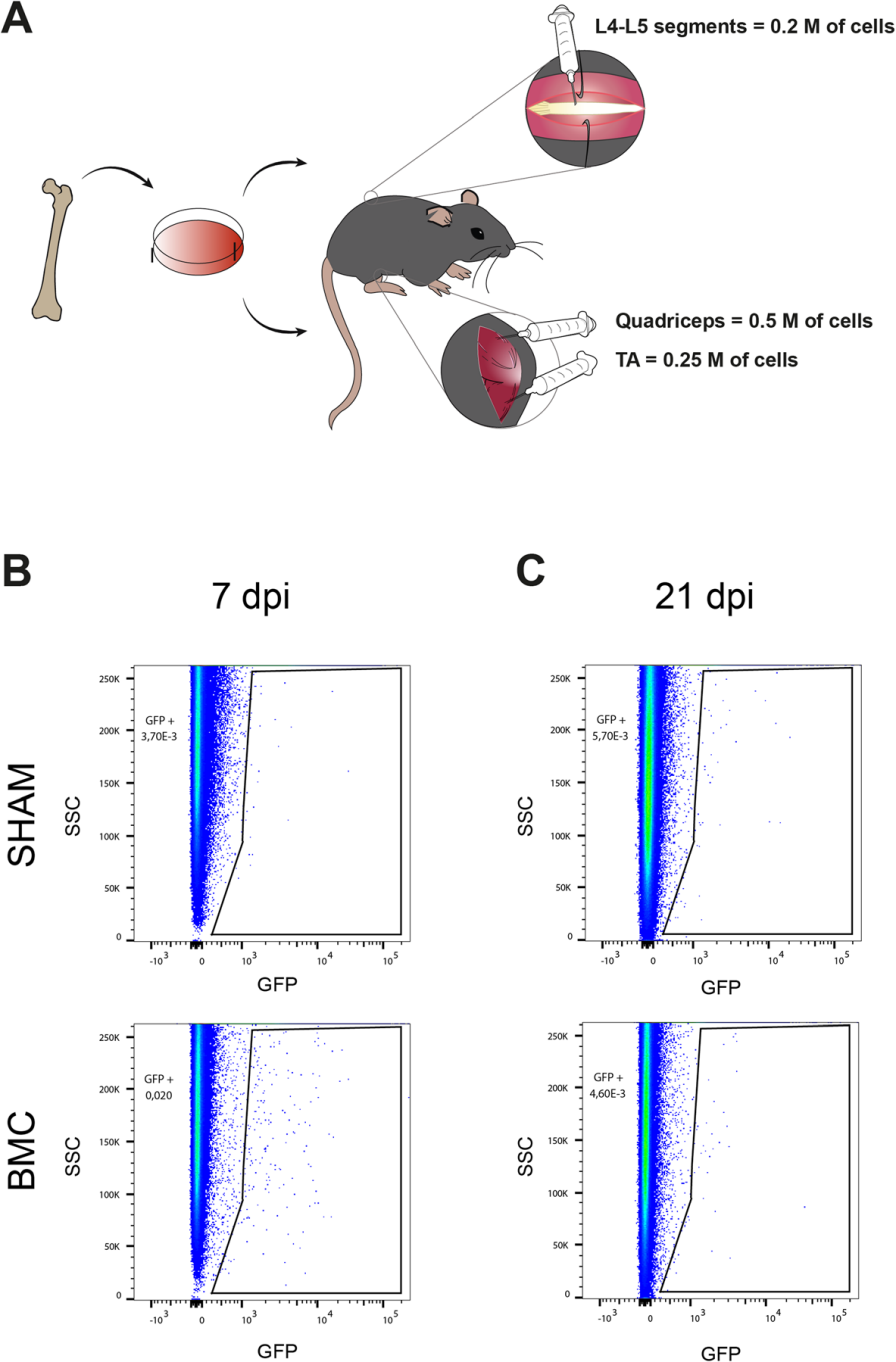
Bone marrow cells survival after injection into the spinal cord of SOD1^G93A^ mice. **a** Illustrative image representing the second therapeutic approach. **b**, **c** Representative dotted FACS plots displaying green-labeled BMCs in the lumbar spinal cord at 7 dpi (**b**) and 21 dpi (**c**) in SOD1^G93A^ mice

To further protect spinal MNs, we injected BMCs into both L4-L5 lumbar spinal cord and hindlimb muscles (Fig. 5a). Firstly, we assessed the survival of the cells after spinal injection. Similar to the muscle injection, BMCs were successfully detected for 1 week after transplantation (Fig. 5b), but they were not present in the spinal cord parenchyma at 3 weeks post-injection (Fig. 5c).

**Fig. 6:**
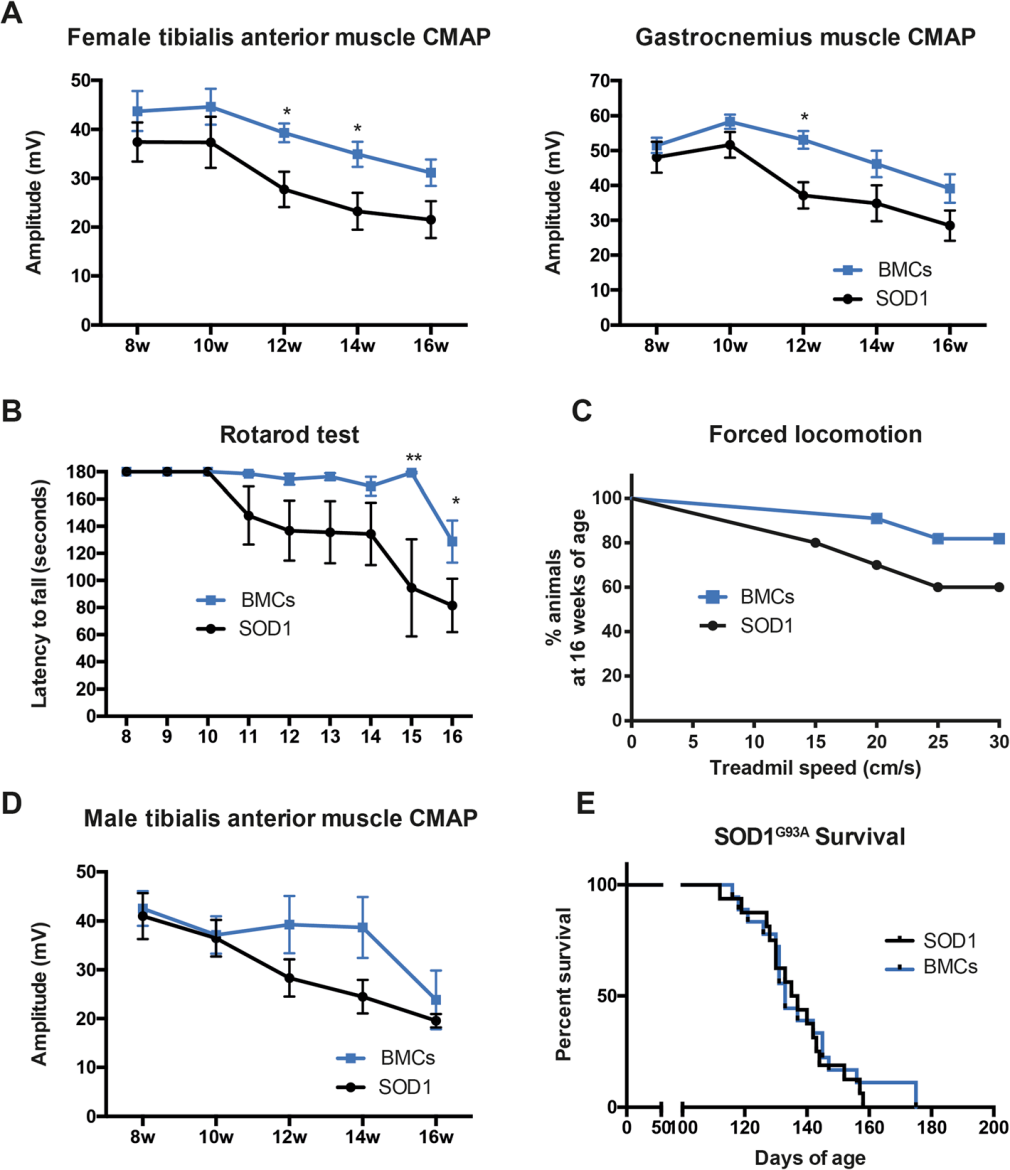
Evaluation of motor outcomes after intraspinal and intramuscular BMC injection in SOD1^G93A^ mice. **a** Electrophysiological evaluation of the compound muscle action potential (CMAP) in BMC-treated muscle (tibialis anterior) and non-treated muscle (Gastrocnemius medialis) in females (*n* = 8 SHAM SOD1^G93A^ vs. 12 BMCs-treated SOD1^G93A^). **b** Evaluation of the locomotion by Rotarod test and **c** forced locomotion in the Digigait (*n* = 10 SHAM SOD1^G93A^ vs. 12 BMC-treated SOD1^G93A^). **d** TA CMAP amplitude in male SOD1^G93A^ mice after BMC treatment (*n* = 5 per group). **e** Evaluation of the maximum lifespan of SOD1^G93A^ mice after combined intraspinal and intramuscular BMC injection (*n* = 16 SHAM SOD1^G93A^ vs. 18 BMC-treated SOD1^G93A^). *p < 0.05 vs. SHAM SOD1^G93A^ mice. Error bars indicate SEM

Despite having a limited survival, we found that the combined intramuscular and intraspinal BMC injection significantly preserved the CMAP amplitude of both treated and untreated muscles, which are innervated by spinal cord-grafted segments (Fig. 6a). Indeed, BMC injection into the spinal cord and muscles further improved motor outcome in SOD1^G93A^ mice as revealed by better performance in the rotarod and forced locomotion tests (Fig. 6b, c). Due to the differences in disease progression between genders [25], we also performed the dual injection of BMCs in SOD1^G93A^ male mice. Similar to female animals, BMC-injected male mice also showed improved CMAP amplitude of TA muscles, although it was not statistically significant, probably due to the small sample size (Fig. 6d). Finally, we assessed the survival of SOD1^G93A^ mice after the combined BMC injection. Since there were no significant differences between genders, we pooled the lifespan of males and females. Even though the combined BMCs-treated animals showed greater preservation of muscular function at early stages of the disease, the treatment failed to increase mice lifespan (Fig. 6e).

### BMC intraspinal and intramuscular injections tend to preserve spinal MNs and to reduce microglial reactivity

**Fig. 7:**
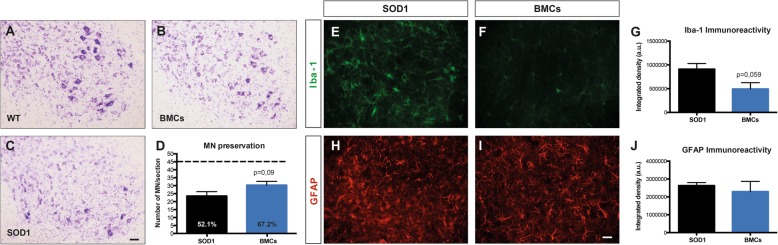
Evaluation of MN preservation and glial reactivity in the lumbar spinal cord after combined BMC injection. **a**, **b**, **c**. Representative photographs of lumbar spinal cord sections showing L4 MNs of **a** WT, **b** BMC-treated SOD1^G93A^ mice, and **c** SHAM SOD1^G93A^ mice. **d** Quantification of MN survival after injection of BMCs in spinal cord and hindlimb muscles. Dotted line indicates the mean number of MNs in wild type mice. **e**, **f**, **h**, **i** Illustrative micrographs showing immunoreactivity against Iba-1 (green, microglia) or GFAP (red, astrocytes) at 16 weeks of age in BMCs-treated SOD1^G93A^ mice and SHAM SOD1^G93A^ mice. Quantification of **g** microglia and **j** astrocytes after injection of BMCs (*n* = 6) or vehicle (*n* = 4) in SOD1^G93A^ mice. Error bars indicate SEM

We then investigated whether the combined BMC injection preserved L4-L5 spinal MNs (Fig. 7a–d). We found that the dual injection of BMCs increased the percentage of MNs spared compared to intramuscular injection (52% intramuscular injection vs. 67% intraspinal and intramuscular injections) but these results were not statistically significant. Since non-neuronal cells actively contribute to MN degeneration, we analyzed the reactivity of astrocytes and microglial cells in the ventral horn of lumbar cord segments. Immunolabeling revealed that the combined BMC injections tend to reduce microglial but not astroglial immunoreactivity in the ventral horn of treated mice (Fig. 7e–j).

## Discussion

The complex pathophysiology of ALS implies a huge difficulty for finding successful therapies. Indeed, most preclinical therapies have failed to translate into human trials due to the wide variety of factors involved in this neurodegenerative disease [9]. For this reason, approaches that simultaneously act on different targets involved in ALS pathophysiology may provide better chances for an effective treatment for patients. Therefore, in this work, we aimed to study different protocols of BMC transplant in SOD1^G93A^ mice. We found that BMCs transplanted into two SOD1^G93A^ hindlimb muscles failed to induce general motor improvement and protection of spinal MNs. However, when these BMC transplants were combined with injection of BMCs into the lumbar spinal cord, the therapeutic effects of BMCs were boosted, resulting in enhanced locomotor skills and a tendency to preserve spinal MNs but did not prolong mice survival.

ALS is a complex neurodegenerative disease where disruption of multiple cellular functions leads to progressive loss of MN. At the central level, MNs die due to the alteration of molecular pathways such as autophagy, aggregation of misfolded proteins, or glutamate-mediated excitotoxicity [8]. However, MN intrinsic pathways are not the only processes involved in MN degeneration. Indeed, different reports point to the contribution of non-neuronal cells, highlighting the importance of glial and immune responses as direct players in the death of MNs [28]. In this sense, peripheral nervous system neuroimmune alterations are also found in ALS [29, 30]. In fact, at the peripheral level, and prior to neuronal degeneration, spinal MNs disconnect from their target muscles in a process known as “dying-back” [23, 30]. As a result, the maintenance of the NMJs is impaired and peripheral axons retract and progressively degenerate. Due to the complexity of ALS disease, which involves events that occur in the central and peripheral systems, as well as in multiple cell types, it is necessary to develop treatments that can target more than one of these pathological episodes.

Cell therapy has emerged as a promising approach to treat multiple disorders due to their potent and diverse beneficial effects [11–13]. BMC transplantation has shown promising results in several animal models of ALS [12, 14, 15, 21]. Since ALS begins with a distal axonopathy that rapidly progresses retrogradely to the neuronal soma, we therefore aimed to first protect the NMJs by transplanting BMCs into the hindlimb muscles at pre-symptomatic stages of the disease. Here we showed that BMCs survived for at least 3 weeks after the injection, which is in accordance with previous reports revealing the presence of BMCs in the muscle tissue between 1 and 2 months after injection [14, 15]. Similar to a recent study [21], we found that intramuscular injection of BMCs did not preserve neuromuscular integrity nor was translated into general improvement of locomotor outcomes. This lack of therapeutic effect may be explained because of several factors. First, we only treated QF and TA muscles, which are not the only effectors involved in locomotion. Second, the limited grafting of the cells could restrict the therapeutic effects to the first weeks after injection. We found that BMCs remained inside the muscle at least for 3 weeks after injections, but they are unlikely to survive for much longer periods. Although in a previous study we showed that some cells were still grafted after 2 months post-injections, the number of cells at that stage was scarce [14]. Therefore, it is expected that greater beneficial effects could be achieved by doing multiple BCM injections over disease progression, as well as, by increasing the number of grafted muscles.

Despite the limited functional improvement observed after intramuscular injection of BMCs, when quantifying the number of MNs in the lumbar spinal cord, we found a tendency to have higher number of surviving neurons in BMC-injected mice. In previous reports, we revealed that BMCs are able to produce GDNF, which can be retrogradely transported to the neuronal soma and may account for the 10% increased MN survival observed after muscle grafting of BMC [14, 15]. However, this beneficial effect was insufficient to induce extensive neuronal sparing. Indeed, the functional improvement found in the previous study in SOD1^G93A^ mice was mild [15], and we have not been able to reproduce it under slightly different conditions, such as age at injection, housing laboratory, and animals colony. A recent report showed that, whereas BMCs injected intramuscularly or intravenously did not produce significant improvements in the SOD1^G93A^ mice, simultaneous injections in the muscle and intravenously delayed the onset of disease and decreased microgliosis although this did not preserve lumbar ventral horn MNs [21], For these reasons, we decided to inject BMCs in the hindlimb muscles and also into the lumbar spinal cord of SOD1^G93A^ mice. We found that, in contrast to the skeletal muscles, the survival of the BMCs within the spinal cord was limited to just a few days. These results are consistent with previous reports indicating that survival of grafted cells in the spinal cord is strongly compromised after 1 week [12, 22, 31]. This is likely due to the immune reaction found within the nervous system of the ALS mice [28]. Since microglial cells become activated at pre-symptomatic stages of the disease, they may rapidly phagocytose the injected cells [29, 32]. Indeed, this immune response may account for the difference in the survival rate of the grafted BMCs in the spinal cord as compared to the skeletal muscle. At peripheral level, infiltration of circulating monocytes occurs even prior to microgliosis [29]. However, they accumulate within the nerve bundles but not at the NMJs or muscle fibers [30]. This less prominent immune reaction in the muscle could increase the survival of BMCs. Despite BMCs having a short-time survival in the spinal cord, injection of BMCs into the lumbar cord and hindlimb muscles improved the global motor function of SOD1^G93A^ mice. This new approach led to ~ 15% greater preservation of spinal MNs at 16 weeks of age as compared to control mice and doubled the beneficial actions observed after transplantation of BMCs into the hindlimb muscles. Even though we cannot rule out the possibility that the beneficial effects found when combining both injections rely only in the intraspinal approach, previous reports support that the combination of different BMC administration routes is needed to reach therapeutic effects in SOD1^G93A^ mice [21]. In line with these findings, other studies have revealed that intraspinal BMC injection in SOD1^G93A^ mice leads to a mild and transitory delay in disease progression [12, 22], strongly suggesting that our findings are likely due to the concomitant effects of the intramuscular and intraspinal injections. This overall therapeutic effect might be explained by the production of growth factors, as well as, by the immunomodulatory effects of the BMCs [21, 33]. Indeed, we found a marked reduction in microgliosis in the spinal cord of SOD1 mice after intraspinal injection of BMCs, even though the results did not reach statistical significance. Previous reports have revealed that microgliosis is a key factor contributing to MN death [29, 34]. Therefore, it is not surprising to observe greater MN survival by reducing microgliosis. Despite the beneficial actions of this dual transplantation, the survival of mice was not prolonged, which is likely due to the protective effects of the BMCs at the site of the transplant (lumbar region) but not in those CNS areas where they were not grafted.

## Conclusion

Overall, our study indicates that combining intramuscular injection of BMCs with BMC transplants in the spinal cord enhanced the therapeutic actions of the cells and improved motor function and preservation of spinal MNs. Although this injection protocol is insufficient to increase mice survival, we do not discard that this could be achieved by repeating the cell injection at different time-points over the disease progression, in multiple muscles and areas of the CNS. Thus, our work suggests that combining muscle and spinal BMC injections represents an interesting cell therapy approach for treating ALS.

## Supplementary information

**Additional file 1: Figure S1.** FACS plots gating and isotype controls. Representative FACS plots showing the gating cut off for each primary antibody.

## Data Availability

The datasets used and/or analyzed during the current study are available from the corresponding author on reasonable request.

## Abbreviations

ALS: Amyotrophic lateral sclerosis
BMC: Bone marrow cells
CMAP: Compound muscle action potential
DMEM: Dulbecco’s modified Eagle medium
dpi: Days post-injection
FACS: Fluorescent-activated cell sorting
fALS: Familial ALS
GFP: Green fluorescent protein
GM: Gastrocnemius medialis muscle
MN: Motoneuron
NMJ: Neuromuscular junction
PB: Phosphate buffer
PFA: Paraformaldehyde
QF: Quadriceps femoris
SEM: Standard error of the mean
SOD1: Superoxide dismutase 1
TA: Tibialis anterior
