# Supplementary figures and images for: Combined intramuscular and intraspinal transplant of bone marrow cells improves neuromuscular function in the SOD1G93A mice
Source: Stem Cell Res Ther. 2020 Feb 7;11:53. doi: 10.1186/s13287-020-1573-6 (PMC7006400; doi:10.1186/s13287-020-1573-6)

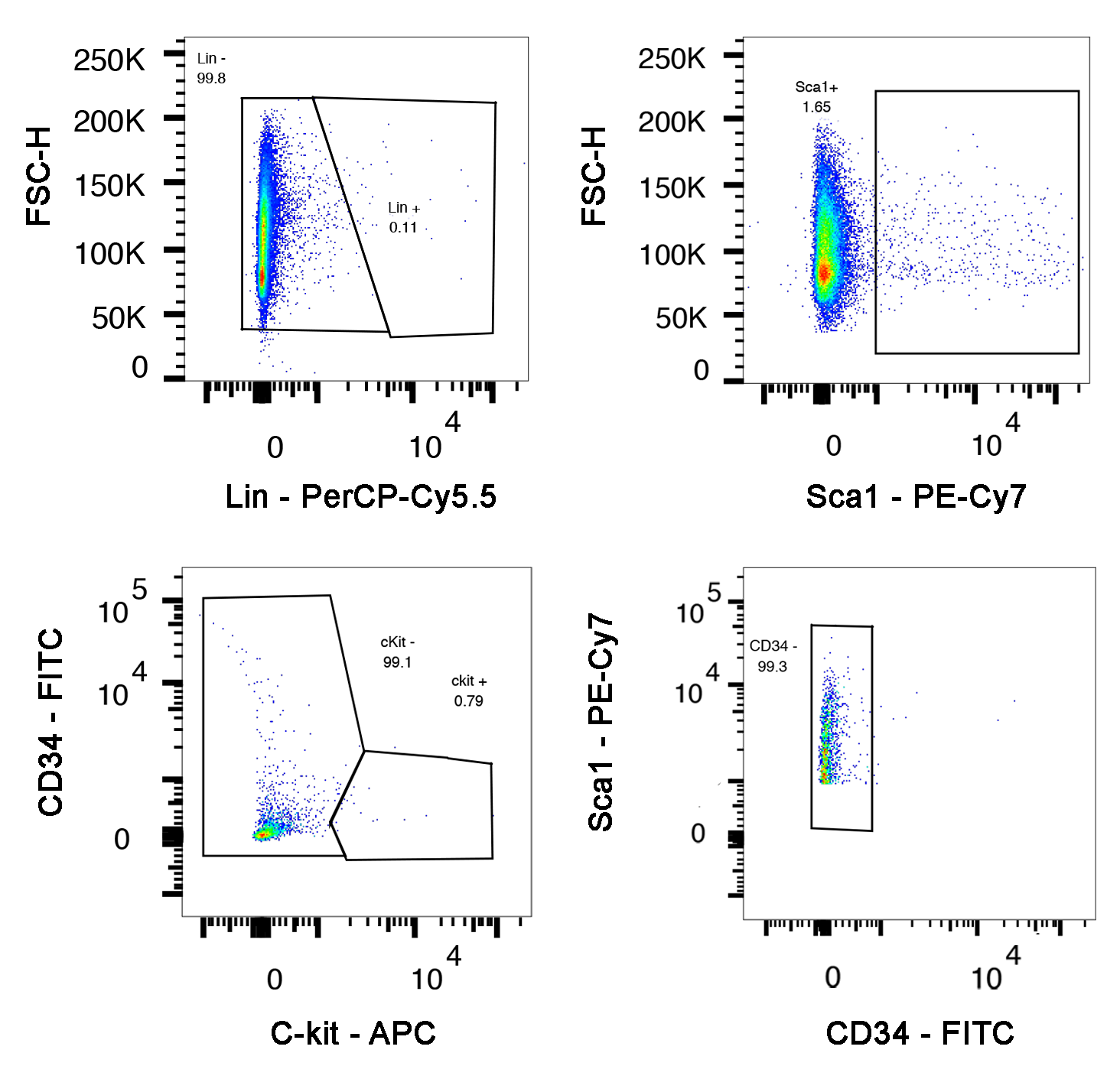

Supplement: Supplementary file 1 — Additional file 1: Figure S1. FACS plots gating and isotype controls. Representative FACS plots showing the gating cut off for each primary antibody. [file 13287_2020_1573_MOESM1_ESM.tif]
